# The relationship between insurance and health outcomes of diabetes mellitus patients in Maryland: a retrospective archival record study

**DOI:** 10.1186/s12913-021-06534-w

**Published:** 2021-05-24

**Authors:** Soo-Hoon Lee, Samuel L. Brown, Andrew A. Bennett

**Affiliations:** Old Dominion University, 1 Old Dominion Way, Norfolk, VA 23529 USA

**Keywords:** Medicare fee-for-service, Medicare managed care, Private insurance, Diabetes mellitus, Length of stay, 30-day readmission, End-stage-renal-disease

## Abstract

**Background:**

Past studies examining the health outcomes of diabetes mellitus (DM) patients found that social determinants of health disparities were associated with variabilities in health outcomes. However, improving access to healthcare, such as health insurance, should mitigate negative health outcomes. The aim of the study was to explore the association between four types of health insurance, namely, Medicare Fee-For-Service (FFS), Medicare Managed Care (MC), Private FFS, and Private MC plans, and the health outcomes of DM patients, controlling for patients’ social determinants of health.

**Methods:**

This is a retrospective cross-sectional archival record study to explore the relationships between types of health insurance and health outcomes of DM patients who were at least 65 years old, or the elderly. Data was drawn from the 2012 Maryland Clinical Public Use Data and received an exempt status from our Institutional Review Board. Elderly Maryland residents with chronic DM were included in the study, resulting in a sample size of 43,519 individuals. Predictor variables were four types of insurance and health outcome variables were length of hospital stay (LOS), 30-day readmission, and end-stage renal disease (ESRD). Control variables included hospital characteristics, patient characteristics, and social determinants of health. Student’s t-tests determined the statistical differences for the control variables between the types of insurance. Multiple hierarchical regression analysis was applied to test the association between insurance plans and LOS, while logistic regression analyses were applied to test the association between insurance plans with 30-day readmission and ESRD. Statistical significance was set at *p* < 0.05.

**Results:**

t-test results indicated minimal statistical differences between the health statuses of patients enrolled in different insurance plans. After factoring out the control variables, regression analyses indicated that Medicare FFS patients had the worst outcome for LOS, 30-day readmission, and ESRD rates. Although patients on Medicare MC plans had lower LOS, 30-day readmission, and ESRD rates compared to those on Medicare FFS, patients enrolled in Private MC plans had the lowest odds of a 30-day readmission and patients enrolled in Private FFS had the lowest odds of an ESRD.

**Conclusions:**

The data suggests that insurance plans were related to the health outcomes of elderly DM patients after considering their social determinants of health. Specifically, DM patients enrolled in managed care and private insurance plans had better health outcomes compared to those on Medicare FFS plans.

## Introduction

Diabetes mellitus (DM) is a chronic illness in which there is no cure and patients require constant access to healthcare. Worldwide, the number of people with DM increased from 108 million in 1980 to 422 million in 2014 [1]. This number is projected to increase to 552 million by 2030 due to population growth, aging, urbanization, and the increased prevalence of obesity and physical inactivity [2].

Although DM patients represented 9.4% of the US population in 2017, they accounted for 24.8% of hospitalizations [3]. Likewise, while the overall 30-day readmission rate of hospitalized patients in the US was 13.9%, the readmission rate for DM patients was 20.5% in 2014 [4]. The social determinants of health among DM patients have been associated with health outcomes. Social determinants, such as socioeconomic status (SES), ethnicity, and other non-medical factors, such as physical and social environments, access to medical services, and social and health policies, influence health and mortality outcomes because benefits of medical advances may be more likely to flow to those with resources that enable them to avoid health risks or adopt protective strategies [5–7]. For example, DM patients who have lower SES are more likely to experience 30-day readmissions than other populations of DM patients [4]. Improving access to healthcare would mitigate negative health outcomes [5]. Hence, having health insurance provides DM patients with access to care to control their disease progression. We explored the association between types of insurance and health outcomes for a sample of DM patients.

In 2018, 91.5% of the U.S. population have health insurance. Private health insurance covered two-thirds, whereas public insurance, administered by the Centers for Medicare and Medicaid Services, covered the remaining one-third of the insured [8]. Within public insurance, Medicare, a federal program, provides health coverage for the disabled and individuals 65 years or older, or the elderly. Medicaid, in contrast, is a state and federal program that provides health coverage for low-income individuals.

Younger enrollees on government and private insurance plans have different health outcomes. For example, in a recent study, Medicare beneficiaries under 65 years old were more likely to be obese compared to those who have commercial insurance [9]. In another study of DM adults, those on Medicare plans were less likely to be treated with new medications to lower their glucose levels compared to those on private insurance plans [10]. Nevertheless, Davis et al. [11] found that Medicare beneficiaries under the age of 65 years were generally more satisfied with their healthcare coverage vis-à-vis those who were covered by private insurance. Medicare beneficiaries reported fewer problems obtaining access to care, greater confidence in their access, and fewer instances of financial hardship due to medical bills [12]. Medicare eligibility for the elderly is associated with increased health insurance coverage, improved health access, and reduced out-of-pocket healthcare expenses for those with chronic illnesses [13]. However, in comparing the health outcomes between patients on Medicare insurance with those on private insurance, Medicare patients were on average unequivocally older than their private insurance counterparts, confounding the results [14]. Little is known about health outcome differences between elderly Medicare beneficiaries with a chronic illness and their counterparts with private insurance. This study seeks to explore if there are differences.

We choose a state with capitation funding to rule out limitations to healthcare access due to financial considerations. We contribute to the literature by clarifying results on the association between types of insurance plans and health outcomes of elderly DM patients where the payment rate to healthcare providers is the same, thus controlling for financial considerations. We hope that by controlling for financial considerations, our study will provide a clearer understanding of how different types of insurance plans are associated with health outcomes for a sample of elderly patients with a chronic illness who need constant access to healthcare.

In both government and private insurance programs, enrollees can choose between fee-for-service (FFS) and managed care (MC) plans. In FFS plans, enrollees face no restrictions in their choice of healthcare providers, whereas those who choose MC plans are restricted to healthcare providers in specific networks [15]. In FFS plans, healthcare providers are paid directly by the insurer for each individual service rendered to a patient. Concerns have been raised that this system has resulted in excessive prescriptions and test orderings [16]. Over-diagnosis has been associated with poorer health outcomes for patients from overtreatment, unnecessary therapies, or psychological suffering during invasive tests [17]. Additionally, since FFS patients can choose multiple healthcare providers for the same illness, fragmentation of care occurs. When different healthcare providers prescribe different care plans, negative health outcomes may result [18]. In the MC plan, a specific amount is established for each insured person when the enrollee receives care from approved providers regardless of the number of procedures undertaken [15, 18, 19]. To minimize the number of procedures, the healthcare provider actively manages the enrollee’s health. MC enrollees with chronic diseases are required to participate in regular preventive visits, which improves health outcomes and the single-point access prevents fragmentation of care and provides continuity of care [19].

Health maintenance organizations, which provide MC plans, generally report lower overall rates of hospitalization, shorter length of stay (LOS), and fewer hospital readmissions [19]. Some argue that the better health outcomes among enrollees on MC plans vis-à-vis FFS plans are the result of selection bias in which MC programs enroll healthier patients. However, recent studies that used the 2010–2016 Medicare Current Beneficiary Survey found no significant differences between the self-reported health statuses and the presence of chronic conditions of Medicare FFS and Medicare MC beneficiaries [20, 21]. Specifically, Park et al. [21] found no significant differences in the health status of Medicare FFS and Medicare MC beneficiaries who have diabetes. Yayac et al. [22] also found no significant differences between Medicare FFS and Medicare MC participants in terms of LOS or hospital readmissions after total hip and knee arthroplasty. Hence, recent studies suggest that Medicare MC beneficiaries are not healthier than those in Medicare FFS plans. In fact, Panagiotou et al. [23] reported that during the period of 2011–2014, Medicare MC beneficiaries had higher risk-adjusted 30-day readmission rates than Medicare FFS for acute myocardial infarction, congestive heart failure, and pneumonia after correcting for under-reported hospital admissions in the Healthcare Effectiveness and Information Set. Likewise, Figueroa et al. [24] reported that Medicare MC beneficiaries had more comorbidities than Medicare FFS beneficiaries and the former were more likely to receive secondary prevention treatments. Park et al. [21, 25] found that Medicare beneficiaries with new diagnoses of diabetes tend to switch from Medicare FFS into Medicare MC plans so that sicker patients have access to a more efficient care delivery system.

## Methods

### Study design, data, and sample

This was a retrospective, cross-sectional archival record study. Data was obtained from the 2012 Maryland Clinical Public Use Data.^1^ Because this is a public data source containing no personal identifiers, the Institutional Review Board (IRB) of our university determined that this study was exempt from IRB review for research purposes (IRB Reference #164798–1).

Results from past studies that include variables on social determinants of health have been confounded by financial considerations in which access and level of care provided to enrollees were related to costs. We choose Maryland as our study site because this state applies the same capitation model for all enrollees in government insurance plans. A federal waiver exempts hospitals in Maryland from the national Medicare fee schedule. As a result, Maryland hospitals are placed under the same payer system to contain cost growth, improve access to care, and implement payment innovations. Both Medicare FFS and MC healthcare providers are paid the same for each enrollee and have the same incentive to attract healthy participants to minimize costs. Consequently, confounds in utilization rates and differences in the restriction of care between Medicare FFS and Medicare MC patients are minimized because the payment rate to healthcare providers in Maryland is the same for both groups of patients [26].

The data included 694,488 patients who were hospitalized in Maryland hospitals in 2012. Among these patients, 85,712 (12.3%) were diagnosed with DM. We limit the DM sample to those who were at least 65 years old who had the opportunity to choose between Medicare and private insurance. We excluded Medicaid enrollees because it is not a federal-only insurance program but involves state partnership. The final sample for analysis included 43,519 elderly DM patients comprising 37,825 Medicare FFS, 2736 Medicare MC, 1926 Private FFS, and 1032 Private MC patients.

### Outcome variables

Three health outcomes were examined in this study. The first was hospital length of stay (LOS) measured in days, which indicates the efficiency and quality of hospital care [27]. Related to quality of care is the 30-day readmission from the indexed DM-related discharge, which was coded as 1 for yes and 0 for no. The third outcome was whether the patient was hospitalized as having end-stage renal disease (ESRD), which was coded as 1 for yes and 0 for no. ESRD is a costly and disabling condition associated with DM [28].

### Control and predictor variables

Three groups of control variables were included in the analyses as they have associations with health outcomes even though they were not the focus of this study. The first group of control variables were related to hospital characteristics where the patient received care. Specifically, hospital size and teaching status have been associated with access to specialists and resources, which influence health outcomes [15]. Hospital size was coded as 1 if the hospital had more than 400 beds and as 0 otherwise. Operating costs increase for hospitals with over 400 beds due to diseconomies of scale [29]. The teaching status of the hospital was coded as 1 if the hospital is an academic teaching hospital and as 0 otherwise. 30-day mortality rate was found to be lower in teaching hospitals compared to non-teaching hospitals [30].

The second group of control variables that were related to health outcomes but were not the focus of this study were the patients’ demographics, behavioral, and biological factors. Age, measured in years, was included because almost 80% of US adults 65 years and older have some form of dysglycemia, which has significant implications for DM [31]. Gender, coded as 1 for male and 0 for female, was included because DM is more common among men [32]. Marital status, measured as 1 for married and 0 otherwise, was included because unmarried individuals tend to have higher health risks than their married counterparts [33]. Smoking, measured as 1 for smoking and 0 otherwise, has been associated with increased risk of Type 2 DM [34]. Asthma, measured as 1 for asthma and 0 otherwise, and tuberculosis (TB), measured as 1 for tuberculosis and 0 otherwise, were included because DM was associated with impaired pulmonary function [35]. Depression, measured as 1 for depression and 0 otherwise, was included because mood has been associated with adherence to medication regimens for glycemic control [36]. Finally, obesity, measured as 1 for obesity and 0 otherwise, is associated with DM because obese individuals secrete resistin, a hormone that causes insulin resistance [37].

The third group of control variables were the patients’ social determinants of health. The first variable was SES. Since the 2012 Maryland Clinical Public Use Data did not provide the patients’ income data, we used the patients’ zip codes to approximate average household income in accordance with common practices in the literature [15]. A second variable was the size of the patients’ residential neighborhood. Patients who live in metropolitan areas, with populations greater than 1 million people, were coded as living in Urban Neighborhoods and were coded as 1 to reflect the degree of congestion and access to community resources and 0 otherwise [15]. Rural areas have fewer physicians per capita than urban areas, which indicate inequitable healthcare access [38]. A third variable was race, measured as two dichotomous variables, White and Black, because race and ethnicity significantly affect health outcomes due to food insecurity, which can affect glycemic control [39]. By controlling for social determinants of health disparities that has traditionally been the result of a lack of insurance access, we limit selection bias associated with financial considerations that include only healthier patients who have access to insurance and healthcare.

Our predictor variables were the four different types of insurance plans: Medicare FFS, Medicare MC, Private FFS, and Private MC insurance plans.

### Statistical analysis

We used Student’s t-test to make three pairs of comparisons to determine the extent to which the profiles of elderly DM patients were different for those enrolled in each type of insurance plan. The first pair of comparison was made between Medicare and private insurance enrollees, the second pair was made between Medicare FFS and Medicare MC enrollees, and the final pair was made between Private FFS and Private MC enrollees. To partial out the effects of the control variables, we used multiple hierarchical regression analysis to isolate the independent association between insurance plans with LOS and used logistic hierarchical regression analyses to isolate the independent association between insurance plans with 30-day readmission and ESRD. Hierarchical regression analysis determines whether insurance plans explained additional variance on the outcome variables, after hospital characteristics, patient demographics, behavioral, and biological factors, and social determinants of health were considered. We entered the data in incremental blocks of information first by hospital characteristics, second by patient demographics, behavioral, and biological factors, third by social determinants of health, and fourth by insurance to separate the various associations with the outcome variables. We report the standardized regression coefficients or beta, which considers the standard errors of the regression coefficients, for multiple regression analysis and Odds Ratio for logistic regression analyses, which indicates the likelihood that an outcome is associated with the predictor variable. An Odds Ratio below 1.0 indicates a likelihood of less than 100% that the predictor is associated with the outcome, whereas an Odds Ratio above 1.0 indicates a likelihood greater than 100% that the predictor is associated with the outcome.

## Results

Table 1 shows the descriptive statistics of the variables. The mean LOS in our sample was 4.45 days, with a standard deviation of 4.18 days, the mean percentage of patients who were readmitted within 30-days after discharge was 15.71, and 5.09% of patients were hospitalized with ESRD.

**Table 1:** Descriptive Statistics

	Mean	Std. Deviation
Hospitals with over 400 beds (%)	32.97	
Teaching hospital (%)	35.47	
Age (years)	76.87	7.813
Male (%)	44.18	
White (%)	63.30	
African American (%)	32.25	
Married (%)	44.85	
Income ($)	$70,103.46	$25,408.93
Urban Neighborhood over 1 m people (%)	60.41	
Smoking (%)	14.69	
Asthma (%)	16.00	
Tuberculosis (%)	8.35	
Depression (%)	10.22	
Obesity (%)	25.37	
Environmental Hazards (%)	0.33	
Medicare Fee-for-Service (%)	86.92	
Medicare Managed Care (%)	6.29	
Private Fee-for-Service (%)	4.43	
Private Managed Care (%)	2.37	
Length of Stay (LOS) days	4.45	4.183
End Stage Renal Disease (ESRD) (%)	5.09	
30-day Readmission (%)	15.71	

*n* = 43,519

Table 2 shows differences between the demographic and medical status profiles of elderly DM patients enrolled in Medicare and private insurance plans, Medicare FFS and Medicare MC plans, and Private FFS and Private MC plans. Table 2 indicates that among this sample of elderly DM patients, there were no statistical differences between Medicare and private insurance enrollees in their health status in terms of smoking (*p* = 0.17), asthma (*p* = 0.29), TB (*p* = 0.24), and obesity (*p* = 0.09). Although the income was significantly higher for this sample of elderly DM patients with private insurance (mean income = $74,624) compared to those with Medicare insurance (mean income = $69,774), the difference was less $5000. Likewise, Table 2 shows that the health status differences between Medicare FFS and Medicare MC enrollees were not significantly different for smoking (*p* = 0.40), asthma (*p* = 0.15), TB (*p* = 0.33), depression (*p* = 0.67), and obesity (*p* = 0.09). The income of enrollees in Medicare MC (mean income = $58,067) was significantly lower than the income of enrollees in Medicare FFS (mean income = $70,624). Medicare MC enrollees were also more likely to live in urban neighborhoods (79.3%) compared to Medicare FFS enrollees (59.3%). Similarly, Table 2 shows that this sample of elderly DM patients enrolled in Private FFS and Private MC enrollees were not significantly different in terms of smoking (*p* = 0.29), asthma (*p* = 0.67), TB (*p* = 0.22), depression (*p* = 0.38), and obesity (*p* = 0.43). In sum, the t-tests results in Table 2 show minimal statistical differences in the health status of elderly DM patients who were enrolled either between Medicare and private insurance plans or between FFS and MC insurance plans.

**Table 2:** Comparison of Enrollee Profiles between Insurance Types

	Medicare	Private	***P***-value	Medicare FFS	Medicare MC	***P***-value	Private FFS	Private MC	***P***-value
Hospitals> 400 beds (%)	32.8	35.1	0.01	31.8	47.3	<.001	35.8	33.9	0.33
Teach hospital (%)	35.3	37.3	0.03	33.9	55.2	<.001	37.8	36.4	0.47
Age (years)	77.22	72.07	<.001	77.25	76.72	<.001	71.95	72.28	0.22
Male (%)	43.23	57.2	<.001	43.5	39.2	<.001	59.1	53.6	<.01
Married (%)	43.53	63.02	<.001	44.3	32.8	<.001	64.8	59.6	<.01
Smoking (%)	14.62	15.55	0.17	14.58	15.17	0.40	16.10	14.53	0.29
Asthma (%)	16.05	15.28	0.29	15.98	17.03	0.15	15.06	15.70	0.67
TB (%)	8.31	8.92	0.24	8.27	8.81	0.33	9.40	8.04	0.22
Depression (%)	10.31	8.99	0.02	10.33	10.05	0.67	9.35	8.33	0.38
Obesity (%)	25.28	26.71	0.09	25.25	25.66	0.63	26.22	27.62	0.43
White (%)	63.32	62.95	0.68	64.8	42.4	<.001	64.3	60.4	0.03
Black (%)	32.41	29.99	<.01	30.8	54.5	<.001	29.1	31.6	0.17
Income ($)	69,774	74,624	<.001	70,624	58,067	<.001	72,776	78,049	<.001
Urban (%)	60.69	56.6	<.001	59.3	79.3	<.001	57.8	54.2	0.06
Environmental Hazard (%)	0.34	0.30	0.78	0.31	0.41	0.34	0.28	0.35	0.38
LOS (days)	4.47	4.12	<.001	4.47	4.51	0.67	4.15	4.05	0.51
ESRD (%)	5.2	3.5	<.001	5.2	5.4	0.63	3.5	3.5	0.99
30-day Readmit (%)	15.9	13.4	<.001	16.0	14.3	0.02	14.3	11.6	0.04
n	40,561	2958		37,825	2736		1926	1032	

We factored out any variations in medical status differences between enrollees in Medicare and private insurance, Medicare FFS and Medicare MC insurance, as well as Private FFS and Private MC insurance by controlling for them using hierarchical regression analyses. By considering the health statuses of elderly DM patients before the insurance variable, we isolated the additional variance explained by insurance on health outcomes. Table 3 shows the hierarchical linear regression results of insurance on LOS after factoring out the control variable pertaining to hospital characteristics, patient demographics, behavioral, and biological factors, and social determinants of health. Table 3 shows that after factoring out the control variables, elderly DM patients who enrolled in Medicare FFS had significantly longer LOS at *p* < 0.01, while those who enrolled in Medicare MC, Private FFS, and Private MC had significantly lower LOS at *p* < 0.05.

**Table 3:** The Relationship between Insurance Type and LOS

	Base Model		Medicare Fee-for-Service		Medicare Managed Care		Private Fee-for-Service		Private Managed Care	
	Beta	*p*-value	Beta	*p*-value	Beta	*p*-value	Beta	*p*-value	Beta	*p*-value
Block 1: Hospital Type										
Hospital with > 400 beds	0.04	< 0.001	0.04	< 0.001	0.02	< 0.001	0.04	< 0.001	0.04	< 0.001
Teaching hospital	0.02	0.02	0.02	0.02	−0.02	< 0.01	0.02	0.02	0.02	0.02
Block 2: Patient Demographics, Behavioral, and Biological Factors										
Age (years)	0.03	< 0.001	0.03	< 0.001	− 0.04	< 0.001	0.03	< 0.001	0.03	< 0.001
Male	−0.01	0.01	0.01	0.92	0.03	< 0.001	0.01	0.96	0.01	0.93
Married	−0.04	< 0.001	− 0.04	< 0.001	− 0.02	< 0.001	− 0.04	< 0.001	− 0.04	< 0.001
Smoking	0.03	< 0.001	0.03	< 0.001	− 0.01	0.30	0.03	< 0.001	0.03	< 0.001
Asthma	−0.01	0.12	−0.01	0.12	−0.03	< 0.001	− 0.01	0.12	− 0.01	0.12
Tuberculosis	0.09	< 0.001	0.09	< 0.001	−0.03	< 0.001	0.09	< 0.001	0.09	< 0.001
Depression	−0.02	< 0.001	−0.02	< 0.001	− 0.01	< 0.01	−0.02	< 0.001	− 0.02	< 0.001
Obesity	0.01	0.13	0.01	0.14	0.11	< 0.001	0.01	0.14	0.01	0.13
Block 3: Social Determinants										
White	−0.02	0.16	−0.02	0.15	−0.06	< 0.001	−0.02	0.15	−0.02	0.15
African American	−0.01	0.62	−0.01	0.59	0.07	< 0.001	−0.01	0.60	−0.01	0.60
Income	−0.01	0.11	−0.01	0.15	−0.01	0.84	−0.01	0.12	−0.01	0.14
Urban Neighborhood	−0.03	< 0.001	−0.03	< 0.001	0.01	0.03	−0.03	< 0.001	−0.03	< 0.001
Environmental Hazards	0.01	0.01	0.01	< 0.01	−0.01	0.27	0.01	< 0.01	0.01	< 0.01
Block 4: Insurance			0.01	< 0.01	−0.01	0.01	− 0.01	0.03	−0.01	0.02

Table 4 shows the hierarchical logistic regression results of insurance on 30-day readmission after factoring out hospital characteristics, patient demographics, behavioral, and biological factors, and social determinants of health. Table 4 shows that elderly DM patients who enrolled in Medicare FFS had a significantly higher likelihood of being readmitted within 30 days (OR = 1.16, *p* < 0.001) while those who enrolled in Medicare MC (OR = 0.88, *p* = 0.03) and Private MC (OR = 0.75, *p* < 0.01) had a significantly lower likelihood of being readmitted within 30 days after discharge from the indexed DM admission. Elderly DM patients who enrolled in Private FFS did not reach a level of significance for 30-day readmission at *p* < 0.05. Thus, the data indicates that this sample of elderly DM patients who enrolled in Private MC had the lowest likelihood among the four types of insurance plans to be readmitted within 30 days while those who enrolled in Medicare FFS had the highest likelihood.

**Table 4:** The Relationship between Insurance Type and 30-Day Readmission

	Base Model		Medicare Fee-for-Service		Medicare Managed Care		Private Fee-for-Service		Private Managed Care	
	Odds Ratio	*p*-value	Odds Ratio	*p*-value	Odds Ratio	*p*-value	Odds Ratio	*p*-value	Odds Ratio	*p*-value
Constant	0.10	< 0.001	0.10	< 0.001	0.11	< 0.001	0.11	< 0.001	0.11	< 0.001
Block 1: Hospital Type										
Hospital with > 400 beds	1.00	0.97	1.00	0.96	1.00	0.94	1.00	0.97	1.00	0.99
Teaching hospital	0.78	< 0.001	0.79	< 0.001	0.79	< 0.001	0.78	< 0.001	0.78	< 0.001
Block 2: Demographics & Medical										
Age (years)	1.00	0.47	1.00	0.75	1.00	0.47	1.00	0.57	1.00	0.647
Male	1.01	0.77	1.01	0.70	1.01	0.77	1.01	0.74	1.01	0.74
Married	0.92	< 0.01	0.93	< 0.01	0.92	< 0.01	0.93	< 0.01	0.93	< 0.01
Smoking	1.11	< 0.01	1.10	< 0.01	1.11	< 0.01	1.10	< 0.01	1.10	< 0.01
Asthma	1.01	0.69	1.02	0.69	1.02	0.69	1.01	0.70	1.01	0.69
Tuberculosis	1.49	< 0.001	1.49	< 0.001	1.49	< 0.001	1.49	< 0.001	1.49	< 0.001
Depression	1.13	< 0.01	1.13	< 0.01	1.13	< 0.01	1.13	< 0.01	1.13	< 0.01
Obesity	1.02	0.55	1.02	0.58	1.02	0.56	1.02	0.56	1.02	0.56
Block 3: Social Determinants										
White	1.77	< 0.001	1.76	< 0.001	1.76	< 0.001	1.76	< 0.001	1.76	< 0.001
African American	1.56	< 0.001	1.57	< 0.001	1.57	< 0.001	1.56	< 0.001	1.56	< 0.001
Income	1.00	< 0.01	1.00	< 0.001	1.00	< 0.001	1.00	< 0.01	1.00	< 0.01
Urban Neighborhood	1.28	< 0.001	1.28	< 0.001	1.28	< 0.001	1.28	< 0.001	1.28	< 0.001
Environmental Hazards	0.88	0.59	0.88	0.59	0.88	0.59	0.88	0.59	0.88	0.58
Block 4: Insurance			1.16	< 0.001	0.88	0.03	0.93	0.25	0.75	< 0.01

Table 5 shows the hierarchical logistic regression results of insurance on ESRD after factoring out hospital characteristics, patient demographics, behavioral, and biological factors, and social determinants of health. Table 5 shows that this sample of elderly DM patients who enrolled in Medicare FFS had a significantly higher likelihood of ESRD (OR = 1.44, *p* < 0.001) while those enrolled in Medicare MC (OR = 0.83, *p* = 0.03), Private FFS (OR = 0.28, *p* < 0.001), and Private MC (OR = 0.58, *p* < 0.01) had significantly lower likelihoods of having ESRD. Thus, the data indicates that this sample of elderly DM patients who enrolled in Private FFS had the lowest likelihood while those enrolled under Medicare FFS had the highest likelihood among the four types of insurance to be hospitalized for ESRD.

**Table 5:** The Relationship between Insurance Type and ESRD

	Base Model		Medicare Fee-for-Service		Medicare Managed Care		Private Fee-for-Service		Private Managed Care	
	Odds Ratio	*p*-value	Odds Ratio	*p*-value	Odds Ratio	*p*-value	Odds Ratio	*p*-value	Odds Ratio	*p*-value
Constant	0.25	< 0.001	0.21	< 0.001	0.26	< 0.001	0.28	< 0.001	0.27	< 0.001
Block 1: Hospital Type										
Hospital with > 400 beds	1.27	< 0.001	1.27	< 0.001	1.27	< 0.001	1.27	< 0.001	1.28	< 0.001
Teaching hospital	0.82	< 0.01	0.83	< 0.01	0.83	< 0.01	0.82	< 0.01	0.82	< 0.01
Block 2: Demographics & Medical										
Age (years)	0.98	< 0.001	0.98	< 0.001	0.98	< 0.001	0.98	< 0.001	0.98	< 0.001
Male	1.33	< 0.001	1.34	< 0.001	1.33	< 0.001	1.34	< 0.001	1.33	< 0.001
Married	0.85	0.001	0.85	0.001	0.85	0.001	0.86	< 0.01	0.85	0.001
Smoking	0.94	0.35	0.94	0.36	0.94	0.36	0.94	0.34	0.94	0.34
Asthma	0.68	< 0.001	0.69	< 0.001	0.68	< 0.001	0.68	< 0.001	0.68	< 0.001
Tuberculosis	0.48	< 0.001	0.48	< 0.001	0.48	< 0.001	0.48	< 0.001	0.48	< 0.001
Depression	0.78	< 0.01	0.77	< 0.01	0.78	< 0.01	0.77	< 0.01	0.77	< 0.01
Obesity	2.58	< 0.001	2.58	< 0.001	2.58	< 0.001	2.58	< 0.001	2.58	< 0.001
Block 3: Social Determinants										
White	0.52	< 0.001	0.51	< 0.001	0.52	< 0.001	0.52	< 0.001	0.52	< 0.001
African American	1.53	< 0.001	1.53	< 0.001	1.54	< 0.001	1.52	< 0.001	1.52	< 0.001
Income	1.00	0.74	1.00	0.63	1.00	0.62	1.00	0.79	1.00	0.84
Urban Neighborhood	1.12	0.04	1.12	0.04	1.12	0.04	1.12	0.04	1.12	0.05
Environmental Hazards	0.57	0.28	0.56	0.27	0.57	0.28	0.57	0.27	0.57	0.27
Block 4: Insurance			1.44	< 0.001	0.83	0.03	0.28	< 0.001	0.58	< 0.01

## Discussion

Past studies reported associations between insurance plans and health outcomes of younger enrollees [9, 10]. This study explored the association between insurance and health outcomes among DM patients who were 65 years or older. The t-tests results for this sample of elderly DM patients showed minimal statistical differences in the health status between those who were enrolled in FFS and MC insurance plans. This lack of statistical differences between MC and FFS enrollees were similar to the reports of Park et al. [21] and Yayac et al. [22]. The mean income of Medicare MC enrollees in this sample of elderly DM patients was significantly lower than those enrolled in Medicare FFS. As well, Medicare MC enrollees were more likely to live in urban neighborhoods compared to Medicare FFS enrollees. The extant literature, which suggests that individuals with lower SES and those who live in urban neighborhoods more likely experience adverse health outcomes, would indicate that the Medicare MC enrollees of this sample of elderly DM patients are not healthier compared to those in Medicare FFS plans, which are in line with the reports of Panagiotou [23] and Figuero [24].

To rule out confounds on health outcomes that arise from hospital characteristics, patient demographics, behavioral, and biological factors, and their social determinants of health, we factored out these confounds in the regression analyses. The data indicates that elderly DM patients who enrolled in Medicare FFS had significantly worse health outcomes in terms of LOS, 30-day readmissions, and ESRD compared to those enrolled in the other three types of insurance. Elderly DM patients who enrolled in Private MC had the lowest likelihood of experiencing a 30-day readmission, while those who enrolled in Private FFS had the lowest likelihood of having ESRD. Private FFS enrollees had lower ESRD compared to Medicare MC or Private MC plans. Thus, the benefits of MC plans were not as strong as those indicated in past studies [18, 19]. Only 30-day readmission risk was lower in MC plans compared to FFS plans.

Given the cross-sectional nature of the data from a single year, we were limited by the ability to determine causality among the factors associated with health outcomes for DM patients 65 years and older. Even though the estimated mean income difference between elderly DM patients on Medicare plans and private insurance plans was less than $5000, the data did not provide information on the employment status of these elderly DM patients. Since the data is publicly de-identified hospital discharge data, collecting follow-up data on patients’ characteristics, such as their employment and other SES data, was challenging. Employment status may be an important factor that influences health outcomes. A recent study indicated that younger diabetic adults under 66 years old were more likely to receive new medications to lower their glucose levels in private insurance plans than Medicare plans [9]. Thus, a future research that explores the health outcomes of elderly DM patients who continue in employment to receive better treatment from employer-sponsored commercial insurance plans would add to the extant literature. Public discharge data also did not record the segmentation of private health plans by socioeconomic categories, which makes it difficult to determine whether high deductible plans were selected by individuals who fall into higher socioeconomic categories because they provide access to Health Saving Accounts with tax advantages.

Nevertheless, given the data restrictions, the results indicate that the selection of insurance choices was associated with health outcomes for a sample of older patients with DM. Future research could mitigate some of these unanswered questions by utilizing longitudinal data. Additionally, a future study could validate the results by examining data for a different chronic illness for which insurance plans and patient characteristics could also play important roles on health outcomes.

## Conclusion

The data suggests that Medicare fee-for-service plans where enrollees in the government insurance plan face no restrictions in their choice of healthcare providers had worse health outcomes in terms of length of hospital stay, 30-day readmission, and end-stage renal disease rates compared to those enrolled in Medicare Managed Care plans or private insurance plans. Public policy could be made to provide incentives for older population with DM to transfer their enrollment to Medicare managed care or private insurance plans. There could even be greater public-private health partnerships with insurance companies and healthcare providers to mitigate the health risks of those with chronic illnesses.

## Data Availability

Data is found in this link: https://hscrc.maryland.gov/Pages/hsp-data-request.aspx

## Abbreviations

DM: Diabetes mellitus
FFS: Fee-for-service
MC: Managed care
LOS: Length of stay
ESRD: End-stage renal disease

## Footnotes

1 https://hscrc.maryland.gov/Pages/hsp-data-request.aspx
